# The Impact of Superplasticizer Chemical Structure on Reactive Powder Concrete Properties

**DOI:** 10.3390/ma18071646

**Published:** 2025-04-03

**Authors:** Stefania Grzeszczyk, Aneta Matuszek-Chmurowska, Natalina Makieieva, Teobald Kupka, Adam Sudoł

**Affiliations:** 1 Faculty of Civil Engineering and Architecture, Opole University of Technology, 48, Katowicka Street, 45-061 Opole, Poland; s.grzeszczyk@po.edu.pl (S.G.); a.matuszek-chmurowska@po.edu.pl (A.M.-C.); 2 Faculty of Chemistry and Pharmacy, Opole University, 48, Oleska Street, 45-052 Opole, Poland; 3 Department of Technical Sciences, University of Applied Sciences in Nysa, 5, Obrońców Tobruku Street, 48-300 Nysa, Poland; adam.sudol@pans.nysa.pl

**Keywords:** reactive powder concrete, superplasticizer chemical structure, concrete properties, density functional theory (DFT)

## Abstract

It is difficult to obtain efficient flowability of reactive powder concrete (RPC) mix due to a low water/binder ratio. The improvement of material flowability could be achieved by using the latest generation polycarboxylate superplasticizers (SPs), as well as by changing the mixing procedure. This paper presents two different superplasticizers’ effect on a fresh mix and hardened reactive powder concrete properties. Results of systematic experimental studies (including physicochemical and spectroscopic tests) and molecular modelling suggest that superplasticizer chemical structure plays a key role in shaping the properties of the concrete mix. It has been demonstrated that SP containing more carboxylate salt groups -COO^−^ Me^+^ improves fluidity of the RPC mix and causes its better deaeration. In contrast, hardened concrete exhibits lower porosity and consequently greater strength. On the other hand, a change in ingredients mixing from a three-stage to a four-stage procedure increased the mix flowability and the RPC strength. The chemical structure of SP and the mixing procedure had no significant impact on cement hydration progress. Our results could be useful both from the point of view of the basic science of materials and the applied field of planning of cement composites in construction.

## 1. Introduction

The progress in concrete technology is closely related to application of mineral additives and chemical admixtures. The effect of fine particles with a size below 10 μm in cement, introduced with mineral additives such as silica fume, fine fly ash and granulated blast furnace slag, as well as limestone powder, is of particular importance. These additives have a significant impact on the materials quality. In this case, modification of rheological properties is particularly important [1,2,3,4,5]. Fine particles addition causes an increase in rheological values especially at a low water/binder (w/b) ratio. Therefore, it is necessary to apply superplasticizers, which in combination with fine particles have a significant effect on rheological properties of concretes [1]. Mineral additives increase the particle packing density in the structure of high-performance cement-composites. In contrast, superplasticizers counteract electrostatic forces and ensure dispersion of fine particles and microfillers reduce viscous forces and friction between aggregated grains. The finer and wider the particle size distribution and the more spherical the microfiller particles are, the better rheological properties can be achieved [4,6]. A commonly used active mineral additive is silica fume. A small size of its particles (10–100 times smaller than cement grains), allows effective filling of spaces between cement grains [7]. According to Bache [8], silica fume is a key factor that increases superplasticizer effectiveness in cement paste. This opinion is also shared by Zang and Han [9] and Kucharska [10]. On the other hand, another research [11] shows that a type of the superplasticizer also affects the SP interaction with a silica fume. The better interaction of the superplasticizer with silica fume in cement was described by higher flowability and has been observed for several other microfillers: finely ground granulated blast furnace slags [5,12,13,14], fly ash [9,15], or limestone powder [1,2,16,17]. Additionally, some studies suggest that fine mineral particles in the presence of the superplasticizer may cause deterioration of rheological properties of cement [1,13]. Achieving fluidity of the cement paste at a low w/b ratio (even below 0.2 in RPC mix), is possible by adding microfillers, which reduces the volume of water required to fill the material voids and increases the excess water to improve fluidity of the cement paste. However, sometimes this procedure is not enough due to the large specific surface area of fine particles. This leads to the formation of water layers on the surface of the microfiller or limits the deflocculation and dispersion of the paste particles by the superplasticizer [6]. For this reason, observed differences in rheological properties of cement pastes containing microfillers are a result of many combined factors. The content of fine particles in cement, their grain size and grain shape composition and used cement type are decisive [18,19].

Other factor, which highly impacts the cement composite properties is the mixing procedure of mix ingredients with water [20,21,22]. This is particularly evident at a low w/b ratio of RPC mixes with fine mineral additives. In general, the RPC mix requires longer mixing times to release water through finer pores [21]. However, it was observed, that homogeneity of the mixture increases up to a certain time point, above which the segregation of ingredients takes place [22]. The RPC mix preparation methodology has not been clearly established yet. Even for the same mix composition, properties of the fresh mix and the hardened RPC vary considerably when the mixing technique, mixing speed and mixing time are changed. The type of a mixer used also has a strong impact on the mixing efficiency and the mix homogeneity [22]. In some papers focused on RPC, different authors have used their own mixing methods with different mixing speeds, duration and an order for dosing ingredients [22,23,24,25,26,27,28,29]. In these studies, the first stage of mix preparation is the dry mixing of ingredients, within 0.5 min to 3 min. Only in one case [28] this time is longer, i.e., 7 min. In two works [24,28], the total amount of water with superplasticizer was added to dry ingredients and mixed within 3–4 min and 10 min, respectively. In other studies, water and superplasticizer were added in two stages, usually 50% of water and 50% of superplasticizer total amount on each time. A larger portion of water (80%, 87%) was also added at the first stage, where the superplasticizer was added at 100% and 50%, respectively [22,25]. The mixing time with the first batch of water and superplasticizer, applied by different authors, ranges from 2.5 min to 9 min, while the mixing time with the remaining amount of water ranges from 6 min to 25 min. The authors [22] compared the effectiveness of a three-stage and a four-stage procedures for preparation of the RPC mix. In the three-step method, ingredients were first dry-mixed, then half the volume of water and half the amount of superplasticizer were added in the second step, and the remaining quantity of water and superplasticizer were added in the third step. In contrast, during the four-stage procedure, cement and silica fume were mixed dry at the first stage, 80% water and 100% superplasticizer were added at the second stage, the remaining mix ingredients, sand and quartz powder were added at the third stage and water (20%) was added at the fourth stage. Based on testing of the concrete microstructure and strength, the more favorable effect of the four-stage mixing procedure was demonstrated. Summarizing the results on RPC mix preparation methodologies, the multi-step mixing method, in terms of its flowability, is a better solution than the one-step preparation. It ensures better wetting of the mix grains at a low w/b ratio resulting in better flowability of the mix [20,21,22]. However, it is difficult to make a definite recommendation for mixing the RPC ingredients on each mixing step, as too many factors affect the properties of the fresh mix and concrete.

A main task during RPC mix preparation is to ensure its flowability. In this case, a superplasticizer combined with fine particles plays a special role. An optimal SP amount, proper dosing time and the ingredients mixing time and speed play a key role in improving the effect of the superplasticizer on the concrete mix flowability. Technologies of state-of-the-art cement-based composite materials, which include reactive powder concretes, require application of more effective superplasticizers, compared to those used in the 1980s, based on sulfonated melamine-formaldehyde resins (SMF) and sulfonated naphthalene formaldehyde resins (SNF) [30]. There are latest-generation polycarboxylate superplasticizers, multifunctional, regular and block copolymers, derivatives of acrylic, methacrylic and maleic acid [31]. A vast majority of currently used more effective superplasticizers are acrylic copolymers. Acrylic copolymers are a wide group of compounds that differ in units` type and their sequence in a polymer chain. Therefore, their effect on the cement pastes fluidity may be different [32]. A mechanism of polycarboxylate superplasticizers action is relatively well known. It is based on their adsorption on cement grains and, apart from anionic electrostatic interactions, in inducing a steric blocking effect by hydrated polyether side chains, which strongly deflocculates cement particles [30]. Superplasticizers used together with microfillers containing fine particles are the basis of a RPC production [30]. However, there are only few data [33,34,35] on the effect of the chemical structure of polycarboxylate superplasticizers on RPC mix flowability. In addition, there is no data on the synergistic contribution of the SP structure and the mixing procedure on the properties of fresh and hardened cement. Therefore, this paper undertakes an examination of the effect of the chemical structure of a polycarboxylate-based superplasticizer on the properties of the mix and the hardened RPC. The research also takes into account the impact of the mixing procedure of the mix ingredients.

## 2. Materials and Methods

### 2.1. Composition of Concrete Mix

The following components were used to prepare the reactive powder concrete (RPC): Portland cement CEM I 52.5 R with a specific surface area of 410 m^2^/kg from WARTA Cement Plant (Działoszyn, Poland), waste silica (0/45 μm) from Łaziska Steel Mill (Łaziska Górne, Poland), quartz powder (0/0.2 mm) and quartz sand (0/0.5 mm) from Osiecznica Aggregate Query (Osiecznica, Poland). Two types of polycarboxylate-based superplasticizer in 30% aqueous solution were used: SP-1 (BASF^®^, Ludwigshafen, Germany) and SP-2 (ATLAS^®^, Łódź, Poland) at 2.5% by weight of the cement, and WHS-12/0.2 steel fibers 12 mm long and 0.2 mm in diameter at 3% by volume. The chemical composition of the cement, silica, quartz powder and quartz sand is given in Table 1. Table 2 shows the grain size composition of components, determined using a Mastersizer 3000 laser grain size analyser (Malvern Panalytical Ltd., Malvern, UK). Cement contained fine particles in a range <5 μm, <10 μm and <20 μm. Cement contained 90% of particles smaller than 37.8 μm, quartz powder contained 90% of particles smaller than 49.1 μm, and for silica it is as high as 250.0 μm. Quartz aggregate contained 90% of particles smaller than 340.0 μm.

To assure the maximum increase of particle packing, concrete mix composition was optimized using Funk and Dinger curve [36]. Four RPC mixes with the same w/b ratio of 0.24, differing in the type of superplasticizer (SP-1, SP-2) were prepared for testing. Composition of RPC mixes is given in Table 3. Cement amount in the concrete mix was 876 kg/m^3^.

### 2.2. Concrete Mixing Procedure

Components were mixed in HOBART HSM20 mixer (Offenburg, Germany) at a speed of 107 rpm. M1 and M2 procedures were used for mixing of RPC components. In M1 procedure (three-stage procedure), in the first stage all components were mixed dry for 1.5 min, then the assumed amount of water was added and mixed for 1.5 min. The next stage was a two-minute interval in mixing. The superplasticizer was in the third stage added and mixed for 10 min. In the M2 procedure (four-stage procedure), the first stage involved dry mixing of cement, silica and quartz powder for 1.5 min. In the second stage, 80% of water amount and the total amount of superplasticizer were added and mixed for 4.5 min. Sand was added in the third stage and mixed for 4.5 min. In the fourth stage, the remaining amount of water (20%) was added and mixed for approx. 4.5 min until the desired consistency was reached. Total time of mix preparation under the three-stage and four-stage procedures was comparable, i.e., 15 min. The M2 procedure involved analogous mixing stages as in the paper [22], but the difference consisted in the change of duration of individual mixing stages and using the same mixing rate for all stages. In both cases, steel fibers were added in the final stage, to the flowing mixture and mixed for 1.5 min. The following RPC mix symbols were used for testing:

- M1 SP-1—RPC mix with SP-1 superplasticizer mixed using three-stage procedure;
- M1 SP-2—RPC mix with SP-2 superplasticizer mixed using three-stage procedure;
- M2 SP-1—RPC mix with SP-1 superplasticizer mixed using four-stage procedure;
- M2 SP-2—RPC mix with SP-2 superplasticizer mixed using four-stage procedure.

### 2.3. Experimental Methodology

The concrete particle size distribution was determined using particle size laser analyser Mastersizer 3000 within the range 0.01–3500 μm.

Tests of RPC consistence were performed in line with PN-EN 1015-3 standard “Methods of test for mortar for masonry—Part 3: Determination of consistence of fresh mortar (by flow table)” [37]. Consistence was determined based on measurement of the concrete flow diameter (fluidic samples were used) with accuracy to 5 mm.

Air content in RPC mix was tested for 1 dm^3^ samples in line with PN-EN 1015-7 standard “Methods of test for mortar for masonry—Part 7: Determination of air content of fresh mortar” [38]. Air content in the mix was determined using a pressure method.

Compressive strength and flexural strength of RPC specimens were tested in line with PN-EN 1015-11 standard “Methods of test for mortar for masonry—Part 11: Determination of flexural and compressive strength of hardened mortar” [39]. Measurements were performed on specimens 40 × 40 × 160 mm.

FTIR spectra of SP-1 and SP-2 superplasticizers were measured in a Thermo-Scientific FTIR spectrometer Nexus (Thermo Fisher Scientific Inc., Waltham, MA, USA) using the transmission method of liquid samples of both superplasticizers. Thin layers of liquid SP were placed on freshly pressed (transparent) KBr pellets. Spectra were recorded in a range from 4000 to 400 cm^−1^.

Determination of the molecular properties of the polymer samples of the tested superplasticizers (weight-average molar masses Mw (g/mol), molar number-average molar masses Mn (g/mol) and distribution of molar masses MWD, MWD = Mw/Mn) was performed by low-temperature gel permeation chromatography (GPC). These analyses were performed using a chromatographic system with a Knauer VWM 275 nm UV reflectance detector (Berlin, Germany). Asahipak GF 310 HQ 300 × 7.6 mm columns (Shodex™, Resonac, Japan) were used and DMF with 0.8% LiCl (DMF = N,N-dimethyl formamide) (Merck, Darmstadt, Germany) was applied as the eluent. These analyses were carried out for 1–2% solutions of the tested superplasticizer in DMF + 0.8% LiCl, maintaining a constant eluent flow through the columns of 0.5 mL/min at 30 °C. The “Grams/386 for Chromatography” software (Thermo Fisher Scientific Inc., Waltham, MA, USA) was used to process the chromatographic data. Standard linear polystyrene references were applied (IUPAC: Poly(1-phenylethane-1,2)) CAS 9008-53-6; -PS500, -PS920, -PS1250 (Merck, Darmstadt, Germany), -PS2850, -PS68000, -PS98300 (Merck, Darmstadt, Germany).

Thermogravimetric measurements were performed using a simultaneous thermal analyser—STA 449 F3 Jupiter (NETZSCH GmbH, Selb, Germany). An analytical sample of approximately 30 mg was introduced into an alumina crucible. The crucible was fitted with a lid to stabilise the atmosphere above the sample during measurement. Once the mass of the specimen had been determined, the crucible was placed on the DSC-TG carrier and the whole was put into the furnace. The temperature programme included specimen stabilization at 30 °C and heating up to 1000 °C at a rate of 10 °C/min.

Testing of RPC porosity was performed using a mercury porosimeter PoreMaster 60 (Anton Paar GmbH, Graz, Austria), within a pressure range from 1 to 400 MPa. Results were presented in a form of differential curves of pore size distribution. Every experiment was repeated in triplicate and all presented results are averaged.

### 2.4. Theoretical Methodology

**Figure 1:**
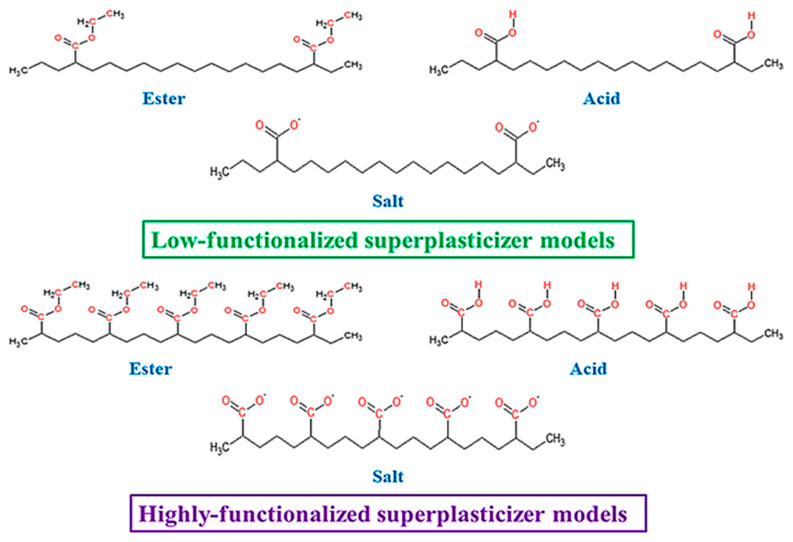
Structures of low- and highly functionalized SP models.

Molecular modelling was performed to support FTIR data interpretation and SP chemical structure effect on cement mix and hardened composite properties description. Gaussian 16 C.01 program was used [40]. Since both analysed superplasticizers SP-1 and SP-2 have a high molecular weight structure, it is difficult to calculate their structural and spectroscopic parameters. In addition, this work considers only contributions of certain functional groups of the superplasticizer. For this reason, simplified models were used that reasonably give the real structure of SP and the contribution of its modifications to the interactions with cement (see Figure 1) [41,42]. All calculations were performed using density functional theory (DFT) in the gas phase. B3LYP hybrid density functional was used due to its efficiency in structural and spectroscopic parameters prediction [43,44,45,46]. Since the models used were quite large, relatively small basis sets were used: 3-21G, 6-31+G* and def2-TZVP [47,48]. In order to make the most accurate analysis of the experimental data using theoretical results, a comparative analysis was performed for all obtained data. The SP models were optimized at all considered levels of the theory. Simultaneously, the IR spectra were simulated. The absence of imaginary frequencies was accepted as a criterion for the equilibrium structure.

## 3. Results

### 3.1. Fresh Concrete

**Figure 2:**
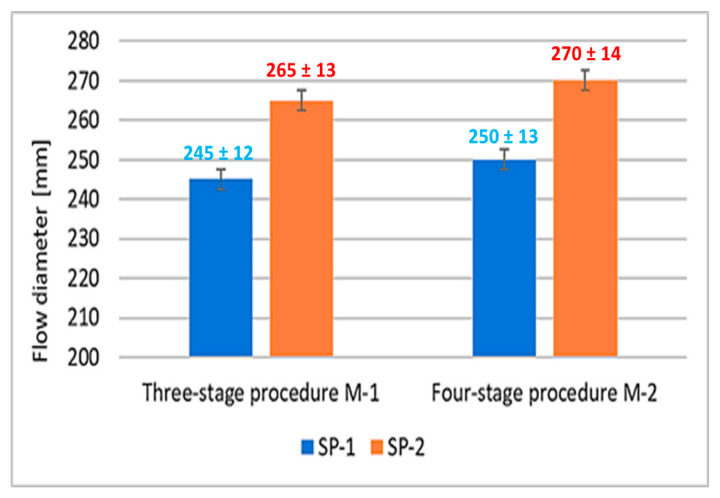
Flow diameter of concrete mix.

Results of concrete mix consistency measurements obtained using two mixing procedures (M1 and M2) and two different superplasticizers (SP-1 and SP-2) by a flow table test method [37], are presented in Figure 2. In this case, the increased flow of the M1 SP-2 and M2 SP-2 mix by about 20 mm was observed, compared to the flow of SP-1 containing mixes. In contrast, changing from a three-stage to a four-stage mixing procedure using both SP-1 and SP-2 increases the fluidity only by ca. 5 mm. It shows that the SP type has a greater effect on concrete mix flow than changing the mixing procedure.

**Figure 3:**
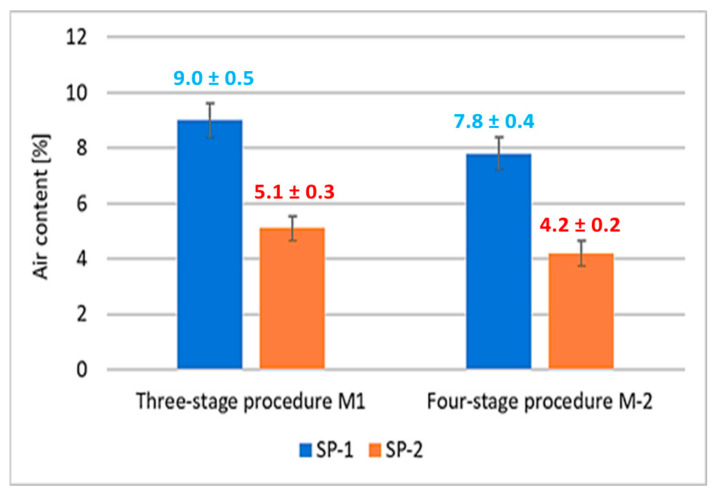
Air content of concrete mix.

The air contents in RPC mixes determined by a pressure gauge method [38], are shown in Figure 3.

It was observed that concrete mixes with SP-2 showed a significantly lower air content by about 45% compared to mixes with SP-1 addition. Whereas, the effect of the mixing procedure on the air content was revealed to a much lesser extent. Changing the mixing procedure from a three-stage to a four-stage results in a 13% reduction in air content in SP-1 containing mixes, and 18% reduction for SP-2 containing mixes. Considering the results of the RPC mix flow tests (Figure 2), it can be concluded that increased degree of RPC fluidity results in reduction of its air content. An analogous relationship between the air content reduction in self-compacting mixes and the increase in their fluidity was demonstrated in [49].

### 3.2. Hardened Concrete

**Figure 4:**
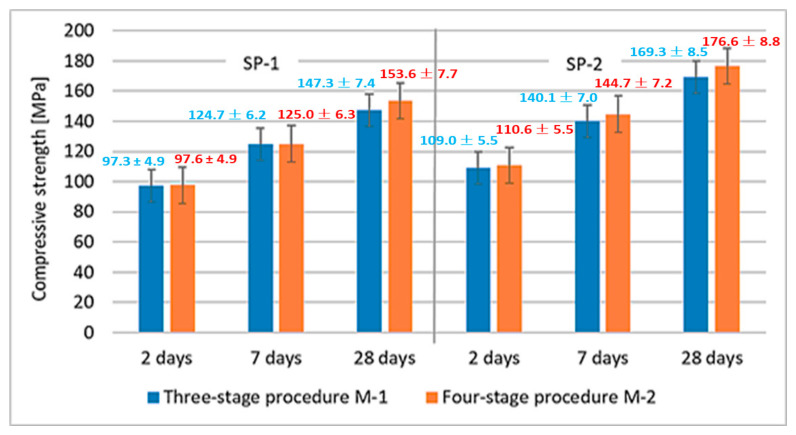
RPC compressive strength after 2, 7 and 28 days.

Results of RPC compressive strength tests are shown in Figure 4. A significantly higher effect of the superplasticizer type used is observed in comparison with the mixing procedure. The compressive strength is higher by ca. 12–16% for M1 SP-2 and M2 SP-2 mixes.

**Figure 5:**
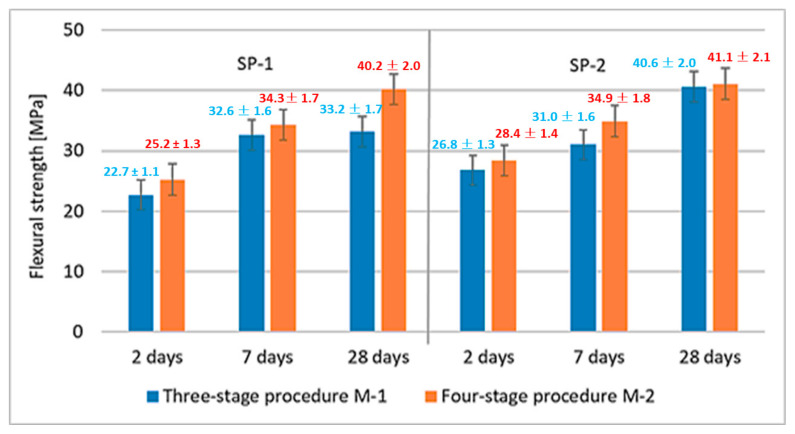
RPC flexural strength after 2, 7 and 28 days.

The effect of M1 and M2 mixing procedures after 2, 7 and 28 days of curing are more or less similar. The largest difference is observed after 28 days, when the compressive strength of RPC, using the four-stage mixing procedure, is by ca. 7 MPa higher. Results of RPC flexural strength tests are shown in Figure 5. Results of RPC flexural strength testing showed that, analogous to the compressive strengths, concretes achieve higher strength values when SP-2 is used (Figure 5). It has been found that application of different mixing procedures for RPC mix (M1 and M2) affects the flexural strength. In this case, using the M2 procedure, provides higher flexural strength values.

### 3.3. RPC Phase Composition and Porosity

#### 3.3.1. X-Ray Powder Diffraction

**Figure 6:**
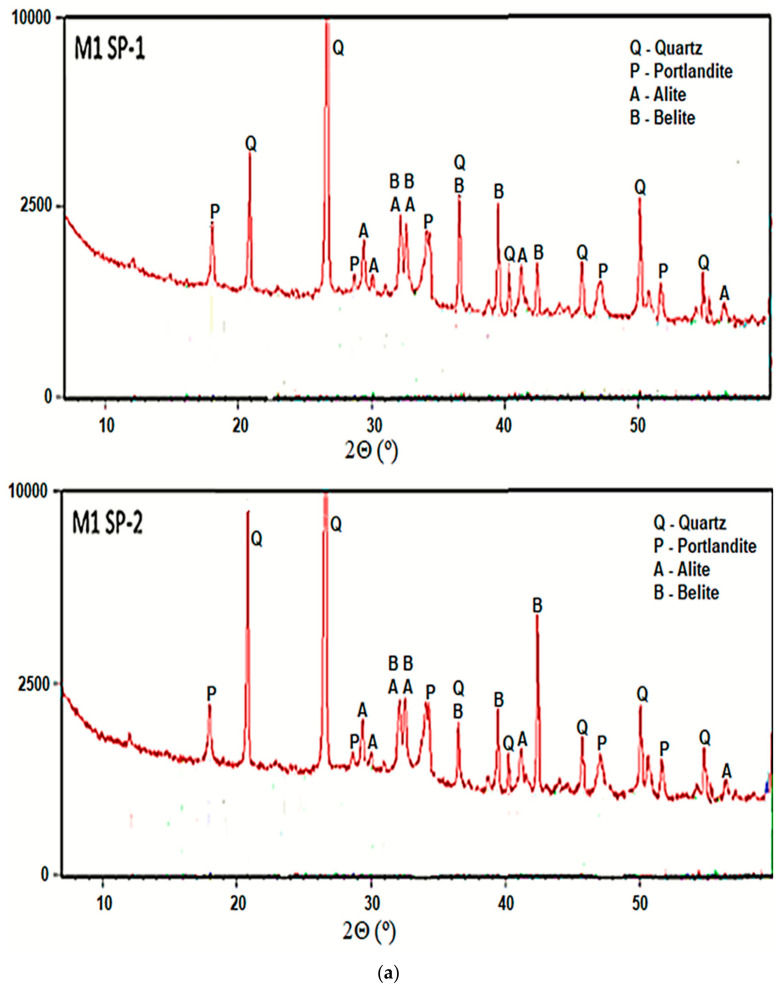

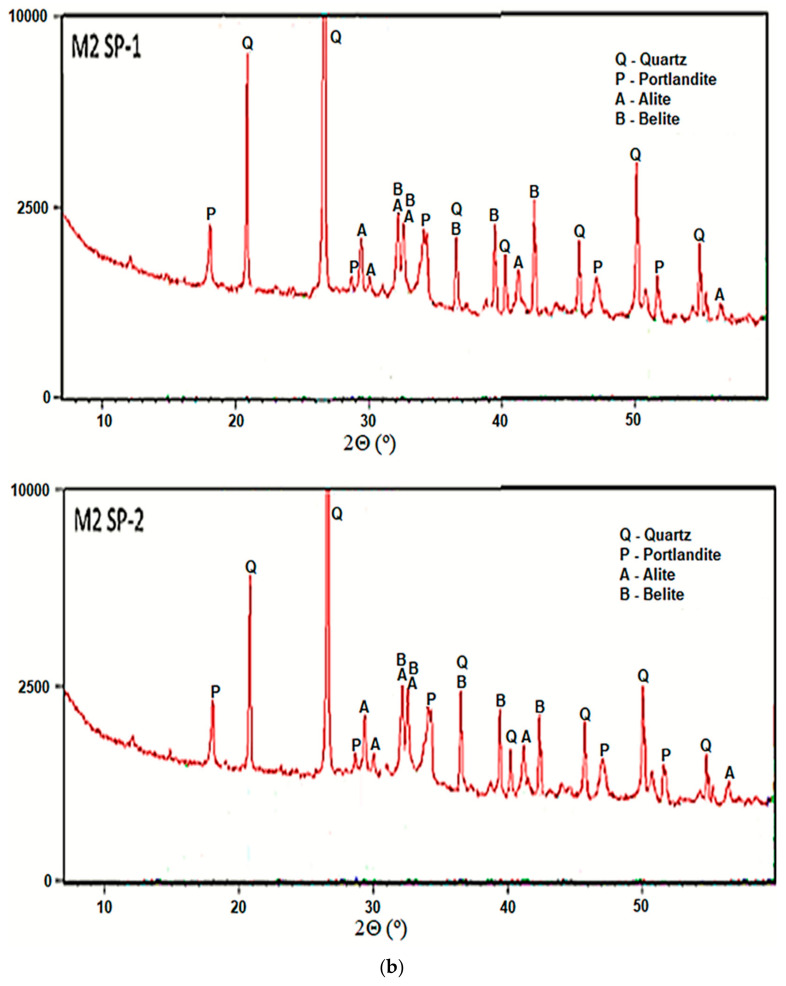
XRD of RPC specimens for RPC mixes with SP-1 and SP-2 obtained (**a**) using M1 and (**b**) M2 procedures after 2 days of curing.

Phase composition of all analyzed RPC composites after 2 days of curing, was determined with the XRD method. Figure 6 shows results of concrete phase composition analyses. As can be seen, all RPC samples show non-hydrated clinker phases (alite, belite). A low w/b ratio it means that up to 50% of the cement grains remain unhydrated. Reflections from cement hydration products, i.e., portlandite (Ca(OH)_2_) are presented. The presence of significant amounts of quartz were also detected in all samples. It is evident that the hydration progress in the tested samples is approximately at the same level, irrespectively of the concrete mixing procedure and the type of superplasticizer used.

#### 3.3.2. Thermogravimetry

**Figure 7:**
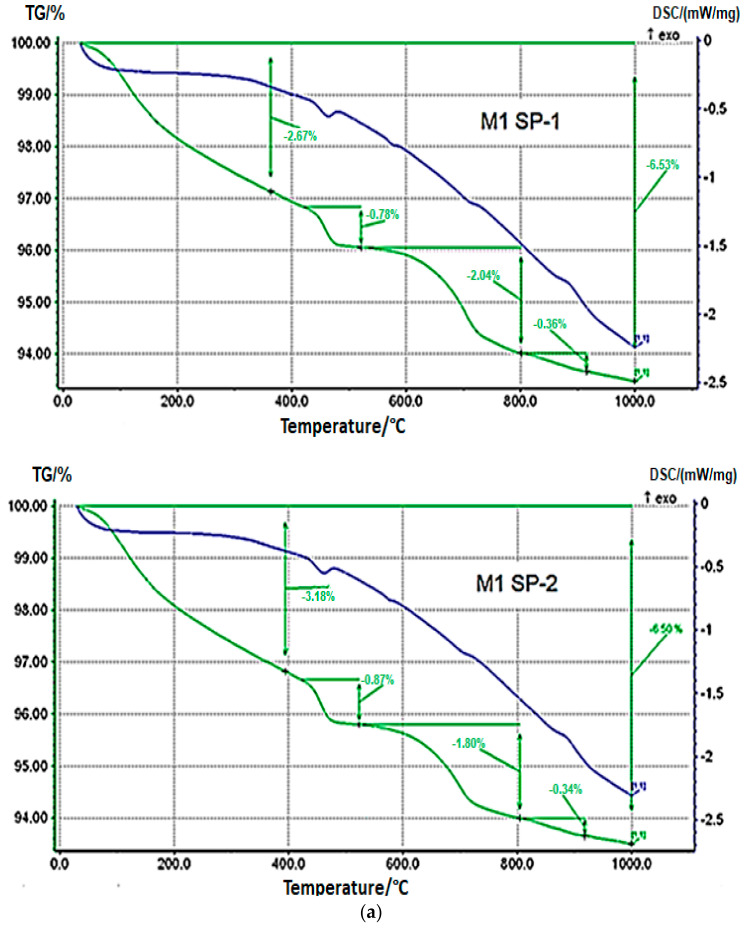

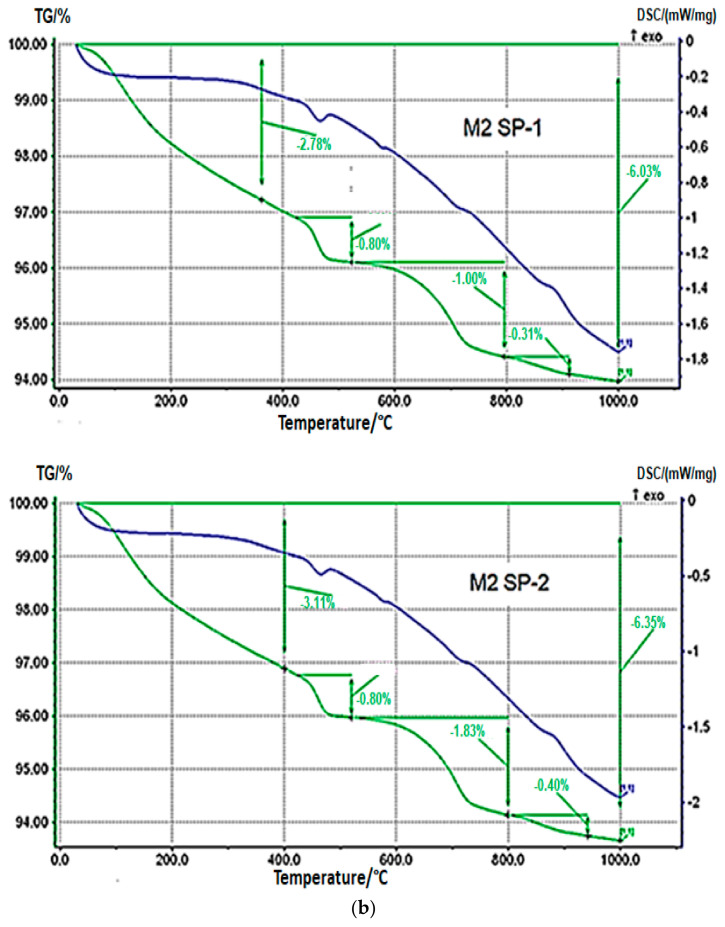
Thermograms of RPC samples (green curves) (**a**) with SP-1 and SP-2 obtained after three-stage mixing procedure (M1) and (**b**) with SP-1 and SP-2 obtained after four-stage mixing procedure (M2) after 2 days of curing.

Results of TGA/DTG measurements for M1 SP-1, M2 SP-1, M1 SP-2 and M2 SP-2 samples after 2 days of curing (Figure 7) confirm the results of XRD phase composition. DTA curves show a weak endothermic effect within a range of 450–470 °C, which is related with portlandite dehydration, and a weak endothermic effect in the range of 750–770 °C, associated with decomposition of calcite. Portlandite (Ca(OH)_2_) content in RPC samples determined based on mass loss and calculated according to [50,51,52] in the temperature range 450-470 °C is small (see Table 4). However, the content of portlandite is slightly higher in case of SP-2 samples (0.87% for M1 SP-2 and 0.78% for M2 SP-2). However, in case of four-stage mixing procedure, the portlandite content is the same for SP-1 and SP-2, and it amounts to 3.29%. Therefore, it could be assumed that the amount of the portlandite phase formed after 2 days of hydration is on the same level, regardless of the type of superplasticizer used and components mixing procedure applied.

#### 3.3.3. Porosity

**Figure 8:**
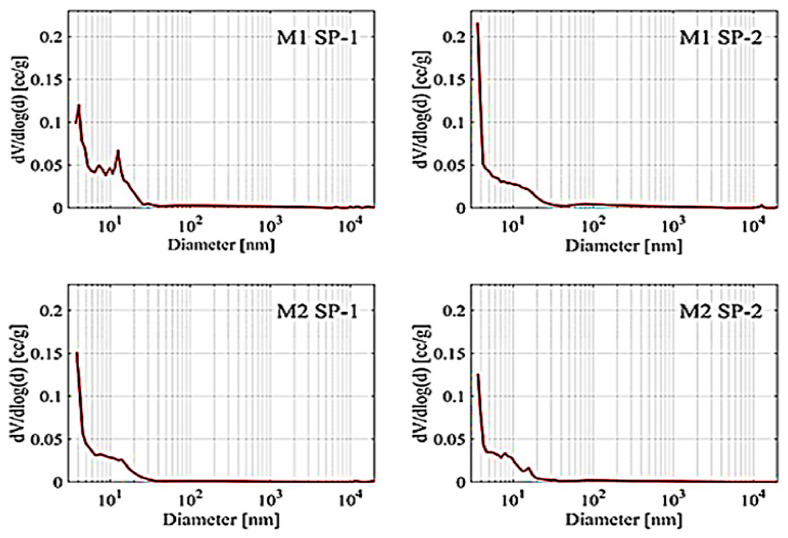
Pore volume distribution vs. pore diameter in the M1 SP-1, M1 SP-2, M2 SP-1 and M2 SP-2 RPC samples after 28 days of hydration.

Results of porosity testing after 28 days, determined by a mercury porosimeter, are presented in Figure 8 and Table 5.

The RPC porosity values showed that SP-2 samples had lower total porosity than SP-1 samples. At the same time, M2 samples demonstrated lower total porosity in contrast with M1 samples. The largest number of pores in RPC tested are <20 nm pores (from 74.3% to 85.9%). Their number was also bigger for M2 samples. The content of larger pores in RPC composites was much lower: from 5.3% to 10.4% for 20–200 nm pores, from 1.5% to 5.5% for 200–2000 nm pores and from 0.4% to 1.5% for 2000–20,000 nm pores. It was observed that the amount of pores significantly decreased with the increase of pore sizes from 20 to 200 nm. A phenomenon of the largest pore content shift towards smaller pores is characteristic for reactive powder concretes [24]. The analysis of the mixing procedure impact on RPC porosity allows us to notice that when the four-stage mixing procedure (M2) is applied, the pore content in the concrete is lower in comparison with the three-stage procedure (M1). Furthermore, by analyzing the content of the largest pores (>20,000 nm) in RPC, it can be concluded that their content is the lowest (3.1%) in the case of M2 SP-2 concrete, i.e., the more effective superplasticizer (SP-2) and the four-stage mixing procedure (M2). On the other hand, for samples prepared using the M1 mixing procedure, the content of these pores ranges between 8.3 and 8.9%.

According to RPC porosity values, it should be concluded that the use of more effective superplasticizer (SP-2) in combination with the four-stage mixing procedure M2 gives the best results in shaping the RPC porosity. Concrete has the highest content of pores < 20 nm and the lowest content of pores between 20 nm and >20,000 nm. Analysis of the total porosity of RPC samples and the pore size distribution has shown that the SP type and the mixing procedure have an impact on the total porosity. The more effective superplasticizer SP-2, which provides higher fluidity of the mix, results in better deaeration of the mix and consequently lower porosity of the concrete. The tests also show that the use of the M2 procedure causes better fluidity of the mix (Figure 2) and reduction in the concrete porosity, compared to the M1 procedure. The four-stage mixing procedure provides a beneficial effect on the pore size distribution in the material. It results in an increase in the pore content of <20 nm and reduction in the number of larger pores in subsequent ranges: 20–200 nm, 200–2000 nm, 2000–20,000 nm. Summarizing, the use of SP-2 and the M2 procedure gives the best effects, ensures high fluidity of the mix. The hardened concrete has the highest number of pores < 20 nm (85.9%) and contains the smallest number of large pores >20,000 nm (3.1%), and furthermore, it achieves the highest compressive strength after 28 days (Figure 4). The authors of the paper [22] also demonstrated that the four-stage procedure was more efficient than the three-stage procedure. It allows obtaining the concrete mix with better fluidity and the hardened concrete with high strength.

### 3.4. FTIR Spectroscopic Analyses of Superplasticizers

**Figure 9:**
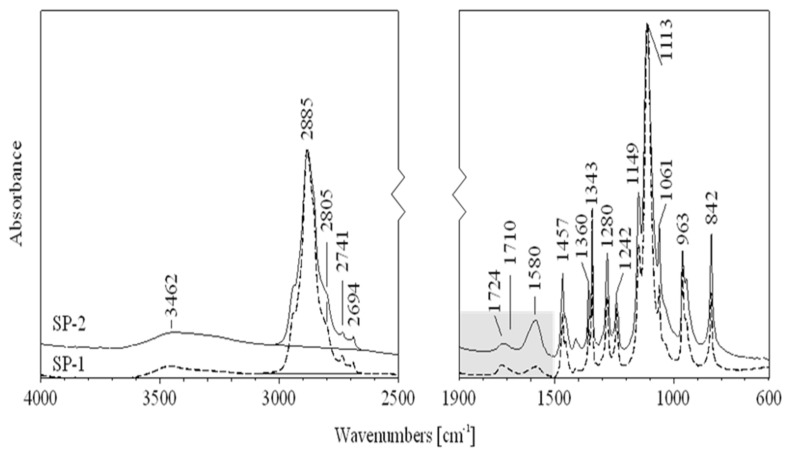
FTIR spectra for SP-1 (dash line) and SP-2 (solid line) superplasticizers.

**Figure 10:**
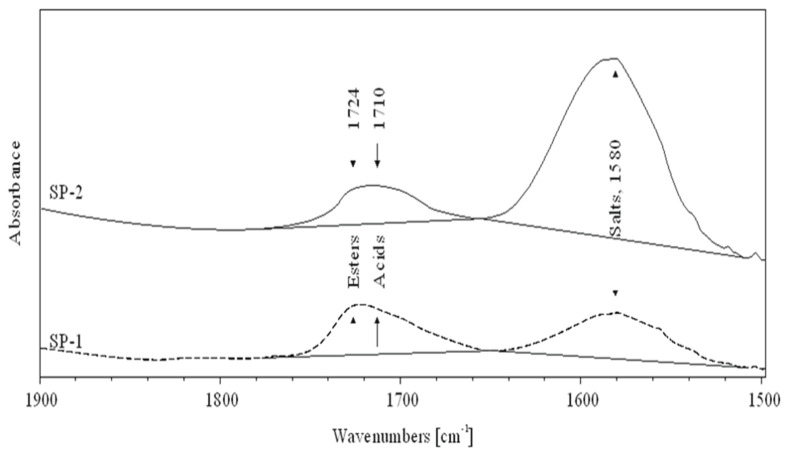
FTIR spectra of SP-1 (dash line) and SP-2 (solid line) superplasticizers in the carbonyl range.

In order to clarify the differences observed in fluidity of RPC mixes, IR spectroscopy and the low-temperature gel permeation chromatography GPC method measurements, discussed in the next section, have been conducted. The IR spectra of superplasticizers SP-1 and SP-2 are shown in Figure 9, while Figure 10 shows a spectral fragment in the carbonyl range. IR spectral analyses of both superplasticizers (Figure 9) show that they contain identical structural fragments of the superplasticizer polymer, but differ in the content of carbonyl groups (esters, acids and salts) (Figure 10). Tentative assignments of absorption bands in SP-1 and SP-2 spectra are presented in Table 6.

**Figure 11:**
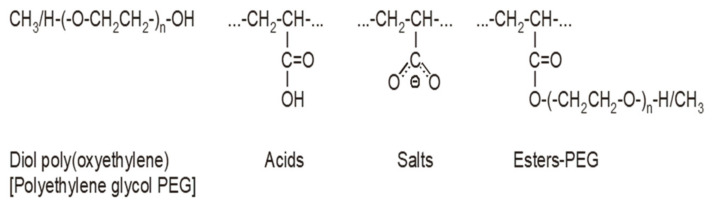
Structural fragments of SPs.

Chemical functional groups present in the SP structure, as listed (Table 6), were identified based on Correlation Charts included in handbooks for FTIR spectrum analysis [53,54,55]. Based on functional groups identified in IR spectra, a general structure of the superplasticizer contains the structural fragments, presented in Figure 11.

Both tested superplasticizers (SP-1 and SP-2) contain almost identical functional groups and structural fragments, but different contributions of individual groups. This is clearly reflected from relative intensities of individual peaks in the carbonyl range (1800–1350 cm^−1^, see Figure 10). Comparison of ester peak intensity (O=C-O-CH_2_- at 1724 cm^−1^) and acid peaks (O=C-OH at 1710 cm^−1^) of both superplasticizers shows that SP-1 contains ca. 50% more ester and ca. 20% more acid groups in its molecules than SP-2, which contains four times more metal carboxylates (salts of R-COO^-^Me^+^ type).

The obtained experimental results are confirmed by the performed molecular modeling data. As can be seen from Table 7, the increase in the SP model functionalization degree leads to the increase in the intensity of the C=O bond stretching in all analyzed functional groups. The opposite trend was observed only once for the salt model calculated at the B3LYP/3-21G level of theory. This can be explained by the small size and low quality of the 3-21G basis set. It can also be noted that at all considered levels of the theory, the results are close to experimental data. This demonstrates that the used model conveyed the spectral properties of the side functional groups of SP quite well. The maximum difference was observed only in the case of the salt model (1660–1630 cm^−1^ compared to experimental value of 1580 cm^−1^). This difference can be partly explained by the choice of incomplete model. Obviously, overestimation by theory of experimental mode frequency is due to omission of unharmonicity in the calculations [56]. A simplified solution to the problem of overestimating C=O mode frequency is to correct it ad hoc by substring −33 cm^−1^ [57]. This action improves significantly the wavenumber of predicted carbonyl stretch. Since the experimental IR spectrum demonstrated the greatest effect of the SP structural modification on the intensity of C=O stretching, attention was focused only on its local chemical environment. Theoretical analysis of ionic bonds in salts requires more extensive computer resources [58]. Therefore, in this case, no significant attention was paid to the position of the peak, but only to its intensity.

It is known that different efficiency could be achieved for polycarboxylate-based superplasticizers, depending on their structure, including the amount of individual functional groups [33,34,35]. The effect of the latest generation polycarboxylate-based superplasticizers is more complex than sulfonated melamine (SMF) and sulfonated naphthalene (SNF) based superplasticizers. It is due to their adsorption on the surface of cement grains and, in addition to electrostatic interactions (between anionic groups preventing cement grains from approaching each other), inducing a steric blocking effect by hydrated polyether side chains, resulting in an even greater fluidity of the paste [30]. Adsorption of the polycarboxylate superplasticizer main chains on the surface of cement grains occurs via carboxylate groups on active centers occupied by Ca^2+^ ions. Anionic groups of salts are responsible for electrostatic anchoring of the superplasticizer molecules to cement grains, and the polyether side chains form a steric blockage around the cement grains, causing their deflocculation [59]. In this case, a relatively small number of these negative anionic groups, contained in the polycarboxylic polymer, contributes markedly to the increased fluidity of the cement paste. Based on comparison of absorption bands of carbonyl groups (Table 8), SP-2 contains over 4 times more carboxylate groups of salts, which explains its greater effectiveness in the RPC mix. The high amount of this structural feature could also be a reason for higher fluidity of the RPC mix containing SP-2 compared to the mix with SP-1. The higher fluidity results in lower content of air in the mix containing SP-2 (Figure 3). In contrast, the lower air content of the mix results in lower porosity of the hardened concrete (Table 5) and higher concrete strength (Figure 4).

### 3.5. Low-Temperature Gel Permeation Chromatography (GPC)

**Figure 12:**
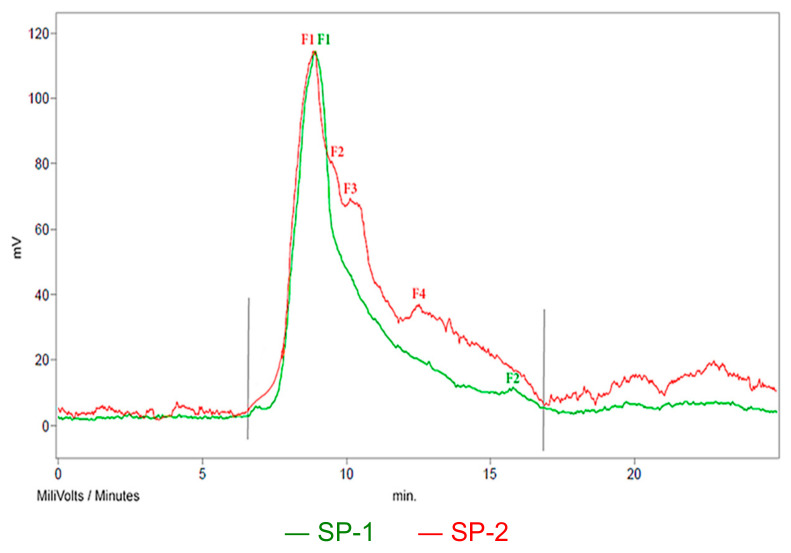
GPC chromatograms with grey limits of the tested superplasticizers.

According to the GPC chromatograms of superplasticizers (Figure 12), the content of individual polymer components in the SP mass and their Mw, Mn and MWD values were determined. The results are presented in Table 9.

The shape of the GPC chromatographic curves (Figure 12) shows that the SP-2 superplasticizer, apart from the main polymer fraction (F1) derived from the polyacrylate copolymer with poly(oxyethylene-PEG), i.e., (poly(PEG acrylate)), contains two other polymer fractions (F2 and F3) in a significant amount (Table 9), derived from free poly(oxyethylene). Fraction F4 is not a polymer; it belongs to a low-molecular substance (Mw = 311 g/mol). The free, unreacted poly(oxyethylene glycol) PEG remaining in the post-reaction system during the synthesis of polyacrylic superplasticizers is not removed from the reaction environment, as it plays a positive role in shaping the properties of the concrete mix. Some studies [60,61,62,63] show that free PEG can cause a positive effect on the process of cement hydration and concrete maturation. Hydrated oxyethylene chains may contribute to the deflocculation of cement grains, which may lead to increased liquefaction of the concrete mix. On the other hand, PEG in water increases the viscosity of the environment due to the hydration of oxyethylene groups; therefore, the leachability of the concrete mix decreases. PEG is also a self-curing concrete agent. In this case, water from the dehydration of PEG polyether chains is used in the cement hydration process in concrete.

According to the GPC results, it could be concluded that the free PEG in the SP-2, constituting about 50% of the polymer fraction, has an undoubted effect on the properties of the concrete mix. According to the manufacturer, the hydrated oxyethylene chains give the concrete mix stability and high fluidity.

## 4. Conclusions

In this work, we showed the importance of the added superplasticizer type (its chemical structure) on the RPC mix fluidity. It was observed that the increased number of salt fragments in the SP increases its efficiency in the RPC mix and resulted in its better fluidity. A consequence of that fact is better deaeration of the mix (the air content in the mix is almost two times lower). On the other hand, application of the four-stage mixing is more effective than the three-stage procedure, as it allows obtaining slightly higher fluidity of the mix and, in consequence, the reduced air content. As a result, a better deaeration of the concrete mix leads to the reduced porosity of the concrete and a subsequent increase in its strength. The RPC compressive strength of SP-2 composite after 28 days is by 23 MPa higher with the M2 procedure and 22 MPa higher with the M1 procedure, compared to the strength of SP-1 concretes. It was observed that the type of the SP plays a key role in shaping the properties of the concrete mix and hardened RPC, while the mixing procedure is less important. No significant effect of the superplasticizer type and the mixing procedure was found on the progress of cement hydration after 2 days. Differences found in the RPC strength when using different types of superplasticizers (SP-1 and SP-2) and different mixing procedures (M1 and M2) are mainly due to a better deaeration of the mix, resulting in reduced porosity, as well as different pore size distribution, leading to the increased strength of the RPC.

## Figures and Tables

**Table 1:** Chemical composition of RPC components (% by mass).

Component	SiO_2_	Fe_2_O_3_	Al_2_O_3_	CaO	MgO	SO_3_	Na_2_O
Cement	21.83	2.00	4.38	65.68	0.93	3.29	0.29
Silica	93.3	2.30	1.50	1.00	- ^a^	- ^a^	- ^a^
Quartz powder	99.00	0.05	0.29	<0.1	<0.1	- ^a^	0.2
Quartz sand	98.60	0.03	0.75	- ^a^	- ^a^	- ^a^	- ^a^

^a^ not analyzed.

**Table 2:** Grain-size distribution of RPC mix components.

Component	Dv(10) ^a^	Dv(50) ^a^	Dv(90) ^a^	Content of Particles		
				<5 μm	<10 μm	<20 μm
	(μm)			(%)		
Cement	2.6	12.7	37.8	26.1	48.4	85.5
Silica	6.8	57.4	250.0	10.9	14.7	26.5
Quartz powder	3.7	18.1	49.1	18.0	34.5	68.1
Quartz sand	118.0	196.0	340.0	-	-	-

^a^ Dv(10), Dv(50), Dv(90)—a particle diameter of the given specimen, below which 10%, 50% and 90% respectively of the material is contained.

**Table 3:** Composition of RPC mixes [% by mass].

Component	Fraction Weight Normalized to the Cement Mass
Cement	1.000
Silica	0.200
Quartz powder	0.120
Quartz sand	1.030
Water	0.240
Steel fibers	0.270
Superplasticizer	0.025

**Table 4:** Portlandite content in RPC specimens after 2 days of curing.

RPC Mix	Mass Loss (%)	Portlandite Content (%)
M1 SP-1	0.78	3.21
M1 SP-2	0.87	3.58
M2 SP-1	0.80	3.29
M2 SP-2	0.80	3.29

**Table 5:** Total porosity and percentage of pores vs. pore diameter in RPC samples after 28 days.

Type of Concrete	Total Porosity (%)	Percentage of Pores (%)				
		<20	20–200	200–2000	2000–20,000	>20,000
		(nm)				
M1 SP-1	8.1	76.5	7.5	4.5	1.5	8.9
M2 SP-1	4.2	83.2	5.3	1.5	0.4	9.2
M1 SP-2	7.2	74.3	10.4	5.5	1.1	8.3
M2 SP-2	3.1	85.9	7.5	2.9	0.5	3.1

**Table 6:** Tentative assignment of absorption bands in SP-1 and SP-2 spectra ^a^.

Wavenumber, cm^−1^		Vibration	Functional Groups and Structural Fragments
SP-1	SP-2		
3462	3442	OH str	-OH hydroxyl groups (alcohols and H_2_O in KBr)
3300-100	3300-3100	OH str	-OH hydroxyl groups (carboxylic acids)
2944	2945	CH_2_ asym str	-CH_2_- (Methylene fragment)
2887	2885	CH_2_(O) sym str	-CH_2_-O- (Methylene groups adjacent to oxygen)
2855	2861	CH_2_ sym str	-CH_2_- (Methylene fragment)
2800	2805	CH str	-CH- (Methine fragment)
2741	2741	CH_2_ scis	1360 cm^−1^ band overtone
2691	2694	2 SCICH_2_	1343 cm^−1^ band overtone
1965	1965	-	
-	-	C=O sym str	O=C-O-C=O (Anhydrides of carboxylic acids)
-	-	C=O asym str	O=C-O-C=O (Anhydrides of carboxylic acids)
1730-1705	1730-1700	C=O str	O=C-(-O-R), O=C-(-OH) (Esters, acids and carbonyl groups)
1577	1581	C=O str	-COO^–^Na^+^ Metal carboxylates
1467, 1455	1467, 1455	CH_n_ def	-CH_3_ and -CH (methyl and methine groups)
1412	1412	-COO^–^ str + OH def	-COO^–^Na^+^, -OH carboxylate salts and hydroxyl groups
1360, 1343	1360, 1343	CH_3_, CH_2_ scis	-CH_2_CH_2_-, (-CH2-O-CH3) methyl and methylene groups in polyethylene fragments
1280	1280	C-O-C str	O=C-O-CH_2_- (Ester groups)
1242	1242	C-C:C-O str	O=C-O-CH_2_- (Ester groups)
1149	1149	C-O-C as str	-CH_2_-O-CH_2_- (Ether fragments)
1110	1113	C-C-O as str	-CH_2_-O-CH_2_- (Ether fragments)
1061	1061	C-O-(H) str	alcohol/ether/ester band
964, 948	963, 947	HC=CH trans wag + rock CH_2_	“trans”- vinylene groups -HC=CH-Ethylene groups -CH_2_-CH_2_-
843	843	C-C-O bend	Oxyethylene groups -O-CH_2_-CH_2_-

^a^ vibration modes: str—bond stretch; sym str—symmetric stretch; asym str—asymmetric stretch; def—angle deformation; scis—scissor; wag—wagging; rock—rocking; bend—bending.

**Table 7:** DFT predicted wavenumbers (ν, cm^−1^) and relative intensities (I) of the C=O stretching in different SP models compared to experimental data (cm^−1^).

Basis Set				Low-Functionalized Model						Highly Functionalized Model								
	Ester		Exp.	Acid		Exp.	Salt		Exp.	Ester		Exp.	Acid		Exp.	Salt		Exp.
	ν	I		ν	I		ν	I		ν	I		ν	I		ν	I	
3-21G	1739	143	1724	1770	196	1710	1668	336	1580	1731	231	1724	1771	317	1710	1660	292	1580
6-31+G*	1783	291		1809	390		1663	491		1784	609		1810	691		1630	518	
def2-TZVP	1780	271		1804	327		1668	533		1780	678		1805	615		1640	794	

**Table 8:** Absorption bands intensity (I) of esters, acids and salts carboxyl groups in SP-1 and SP-2.

Superplasticizer	I		
	Esters	Acids	Salts
SP-1	0.00947	0.00785	0.00812
SP-2	0.00631	0.00631	0.03201

**Table 9:** GPC results of superplasticizer samples.

SP	Polymer Fraction	Content of Polymer Fraction (%)	Molecular Weight (g/mol)		MWD
			Mw	Mn	
SP-1	F1	95.39	13,744	1284	10.71
	F2	4.61	96	72	1.33
SP-2	F1	48.84	23,623	11,491	2.05
	F2	14.22	3588	3481	1.03
	F3	36.94	1636	1418	1.15

## Data Availability

The original contributions presented in this study are included in the article. Further inquiries can be directed to the corresponding authors.
